# Disruption of Four Kinesin Genes in *Dictyostelium*

**DOI:** 10.1186/1471-2121-9-21

**Published:** 2008-04-22

**Authors:** Dilip K Nag, Irina Tikhonenko, Ikko Soga, Michael P Koonce

**Affiliations:** 1 Division of Molecular Medicine, Wadsworth Center, Albany, NY, 12201-0509, USA; 2 Department of Biomedical Sciences, School of Public Health, University at Albany, Albany, NY, 12201-0509, USA

## Abstract

**Background:**

Kinesin and dynein are the two families of microtubule-based motors that drive much of the intracellular movements in eukaryotic cells. Using a gene knockout strategy, we address here the individual function(s) of four of the 13 kinesin proteins in *Dictyostelium*. The goal of our ongoing project is to establish a minimal motility proteome for this basal eukaryote, enabling us to contrast motor functions here with the often far more elaborate motor families in the metazoans.

**Results:**

We performed individual disruptions of the kinesin genes, *kif4, kif8, kif10*, and *kif11*. None of the motors encoded by these genes are essential for development or viability of *Dictyostelium*. Removal of Kif4 (kinesin-7; CENP-E family) significantly impairs the rate of cell growth and, when combined with a previously characterized dynein inhibition, results in dramatic defects in mitotic spindle assembly. Kif8 (kinesin-4; chromokinesin family) and Kif10 (kinesin-8; Kip3 family) appear to cooperate with dynein to organize the interphase radial microtubule array.

**Conclusion:**

The results reported here extend the number of kinesin gene disruptions in *Dictyostelium*, to now total 10, among the 13 isoforms. None of these motors, individually, are required for short-term viability. In contrast, homologs of at least six of the 10 kinesins are considered essential in humans. Our work underscores the functional redundancy of motor isoforms in basal organisms while highlighting motor specificity in more complex metazoans. Since motor disruption in *Dictyostelium *can readily be combined with other motility insults and stresses, this organism offers an excellent system to investigate functional interactions among the kinesin motor family.

## Background

*Dictyostelium discoideum *is a compact amoeba that spends much of its natural existence crawling through the soil, searching for and ingesting bacteria. When food sources are exhausted, individual amoebae trigger a developmental program that initiates both inter and intracellular signaling, to aggregate ~100,000 amoebae and form a multicellular mass. Each cell within this mass undergoes multiple adhesions and conformational changes, forming a cooperative slug that can migrate to new areas. The slug undergoes further multicellular differentiation to form supportive stalk cells, a rudimentary immuno-like surveillance system, and regenerative spores that resist environmental stresses. This dualistic life cycle and its associated transitions (single cell to metazoan organism) have made *Dictyostelium *an attractive model in which to study cell motility, signal transduction, and a relatively simple developmental program (reviewed in [1], see also [2]).

Motility-wise, *Dictyostelium *behaves in a manner similar to that of many vertebrate cells (crawling, sensing, and engulfing targets, robust intracellular movements). Yet, this organism clearly retains a simplicity associated with its relatively small and compact genome, and exhibits features commonly seen in protozoa and fungi (for example, an intranuclear spindle for cell division). Characterization of the actin cytoskeleton in *Dictyostelium *has led to the identification of actin binding proteins, multiple myosin motors, and signaling cascades whose functions are conserved among eukaryotic cells. Preliminary characterization of the microtubule-associated network has revealed a level of complexity intermediate between some of the simple single-celled eukaryotes and metazoans. For example, the machinery in *Dictyostelium *that drives movement along microtubules contains 14 motors (13 kinesin ATPases, 1 dynein ATPase, [3,4]); twice the number found in *Saccharomyces cerevisiae *[5], but less than a quarter of the number encoded in the human genome [6]. Paradoxically, deletions of kinesins whose homologs are essential for vertebrate activities have produced relatively mild phenotypes in *Dictyostelium*. Are these results reflective of *Dictyostelium's *unique life cycle? Or do they reveal core functional redundancies and interactions that, like the actin system work, can be utilized to understand microtubule-based motor action in more complex systems?

**Figure 1:**
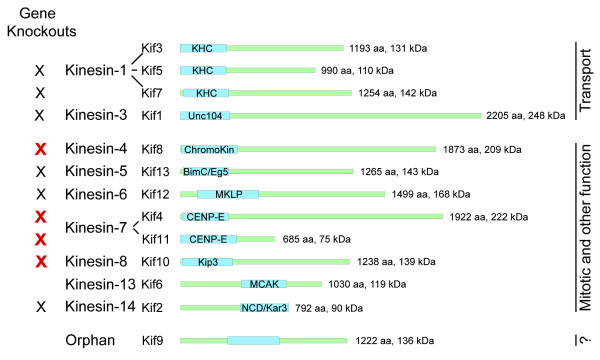
**Kinesin Gene Family in *Dictyostelium***. Schematic representation of the 13 kinesin motors, identified by functional and/or sequence analyses (adapted from ref [3]). The motor domain is indicated in blue, along with the common family name. The remaining neck/linker/tail domains are drawn to scale in green. Both formal and *Dictyostelium*-specific gene names are listed. Preexisting gene knockouts for six of the kinesins are indicated by the black X's (*kif5, kif7, kif1, kif13, kif12, kif2*, [7, 10, 12-14, 46]): new gene disruptions reported in this paper are indicated by the red X's.

Because of their homologies to motors with known functions, 12 of the 13 kinesins in *Dictyostelium *can be placed within existing kinesin families and subdivided into two functional categories (Fig. 1). Four members are closely related to kinesins in metazoans that have organelle transport functions, while eight members are related to motors significant for mitotic events. To date, six individual kinesins have been genetically disrupted by homologous recombination in *Dictyostelium *(*kif's 1, 2, 5, 7, 12, 13*), producing varied effects on cell function. Disruption of *kif12 *(kinesin-6; MKLP family) resulted in significant cytokinetic defects [7,8]. *kif12*^- ^cells failed to divide in suspension, but were able to undergo non-mitotic cytofission on a surface to enable their propagation. For each of the other five kinesins, single deletions did not produce significant effects on cell development or viability. However, closer examination revealed defects suggestive of redundant or cooperative effects with other motor activities. For example, *kif1*^- ^cells (kinesin-3, Unc104) showed a 62% decrease in overall organelle movements *in vivo *[9]. There was a 90% reduction in plus end-directed motility as measured in an *in vitro *assay, but no demonstrable effects on mitochondrial movement. When examined in detail, *kif13*^- ^cells (kinesin-5, BimC/Eg5) showed an increased rate and decreased stability of mitotic spindle elongation [10]. When combined with an otherwise viable dynein perturbation (380 K, [11]), *kif13*^-^/380 K cells were unable to divide properly. *kif2*^- ^(kinesin-14, ncd/kar3), *kif5*^- ^(kinesin-1, KHC), and *kif7*^- ^(kinesin-1, KHC) cells showed mitotic, actin-filament, and developmental defects respectively, but only when challenged with overexpression or competition assays that further stressed the individual cells [12-14]. In contrast, mammalian homologs of *kif12, kif13, kif2 *(and *kif4, kif8, kif10 *in this report) were found to be essential for cell viability [15]. Thus the non-lethal disruption of these genes in *Dictyostelium *offers us the opportunities to examine basal motor activities and interactions, to further understand the motors functions and regulation. We address here the consequences of individual disruption of four kinesin genes in *Dictyostelium*, and we contrast the functional redundancies among such motors in single-celled organisms with their functional specificity in metazoan organisms.

## Results

### *kif4*, *kif8*, *kif10*, and *kif11 *are Not Essential Genes in *Dictyostelium*

**Figure 2:**
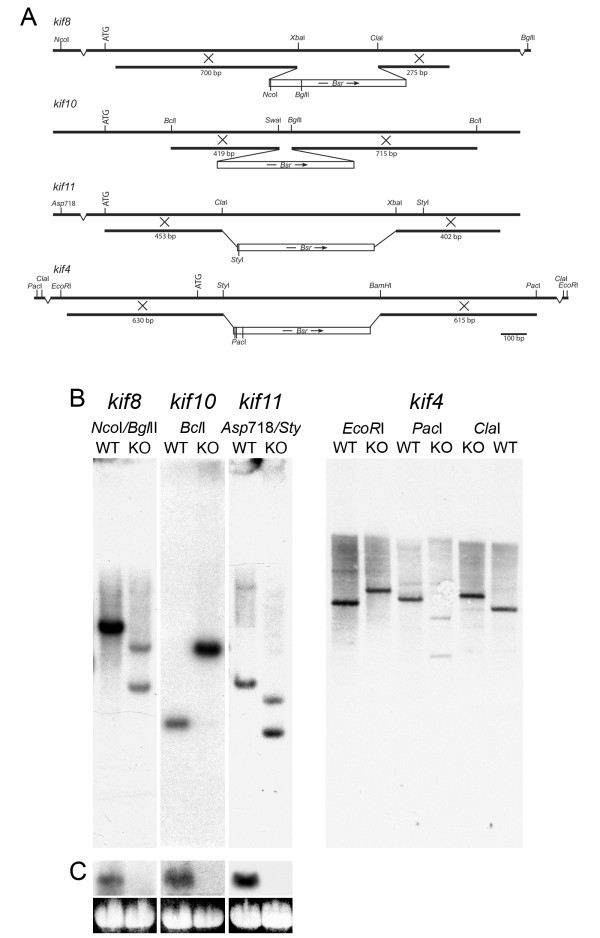
**Kinesin Gene Disruptions**. **(A)**. Schematics of constructs, showing details of the homologous regions and relevant enzymes used to target recombination and to confirm disruption. The position of the *bsr*^*r *^cassette used for selection is also shown. ATG indicates the start of the protein-coding region. **(B)**. Southern blot comparisons of wild-type AX-2 control (WT), *kif8*, *kif10*, *kif11*, and *kif4 *knockout (KO) DNAs. DNA was digested with the indicated enzymes and probed with the entire amplified kinesin gene fragment. Since we were unable to detect kif4 mRNA in wild type cells (see text), we include multiple digests in this panel to demonstrate disruption. All resulting DNA fragments are as predicted from the wild-type and recombination sequences. **(C)**. Northern analysis of AX-2, *kif8*, *kif10 *and *kif11 *knockout cells. Top panel shows mRNA hybridization, bottom panel shows a loading control (4.1 kb 26S rRNA). Note the abundant level of kinesin message in wild-type cells, but the complete absence of message in the disrupted clones.

Genomic fragments of *kif4, kif8, kif10*, and *kif11 *were isolated by PCR amplification of wild-type AX-2 cell DNA, and were used to generate deletion-mutant alleles for their respective kinesin genes (Fig. 2A, Methods). A similar strategy was followed for each gene. Internal restriction enzyme sites of the amplified fragments were utilized to replace coding sequence with a 1.6-kb blasticidin resistance cassette. Genomic sequences flanking the cassette (275–763 bp) targeted the insertion of these mutant alleles into their wild-type gene by homologous recombination, and thus disrupted transcription of the native gene product. Integration of the mutant alleles at their correct sites was confirmed by PCR and Southern blot analyses (Fig. 2B). Northern blot analysis further confirmed loss of full-length mRNA in *kif8*^-^, *kif10*^-^, and *kif11*^- ^transformants (Fig. 2C). There was no evidence for shorter transcripts that would indicate partial expression of the sequence upstream of the integration site for these three clones. Northern blots of *kif4*^- ^were performed, but we were unable to detect the native message in wild-type cells. Very low message levels of the kif4 gene, undetectable during log phase growth has been previously reported [12], and is consistent with tight cell-cycle regulation of kinesin-7/CENP-E homologs in other organisms [16,17].

**Figure 3:**
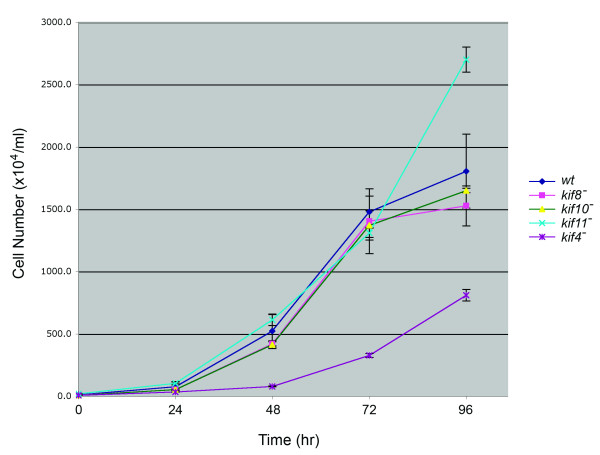
**Comparison of Growth Rates**. *Dictyostelium *enters stationary phase at ~1–3 × 10^7 ^cells/ml, a point reached here after 4 days of growth. Wild-type, *kif8*^-^, *kif10*^-^, and *kif11*^- ^cells exhibit very similar rates of logarithmic phase growth, with a doubling time in axenic medium of 8–9 hrs. *Kif4*^- ^cells grow significantly more slowly than any of the three kinesin nulls as well as the wild-type control. *Kif11*^- ^cells appear to delay their entry into stationary phase (96 hrs), but remain within the normal range of maximum cell density reported for *Dictyostelium*.

Individual disruptions of all four kinesins resulted in viable cells with no gross morphological defects. Cells were comparable in size with wild-type controls and retained the ability to undergo a complete developmental cycle so as to generate viable spores (data not shown). *kif8*^-^, *kif10*^-^, and *kif11*^- ^cells grew at log-phase rates indistinguishable from wild-type AX-2 cells (Fig. 3). Interestingly, *kif4*^- ^cells grew significantly more slowly than the wild type, or the other three kinesin-null strains. This reduced growth rate was observed both in solution (Fig. 3) and on solid support in Petri dishes. *kif11*^- ^cells appeared to reach stationary phase at a higher density than the other strains, but otherwise showed no difference in viability or morphology than wild type cells.

### Microtubule Distributions Appear Normal in Kinesin Null Cells

**Figure 4:**
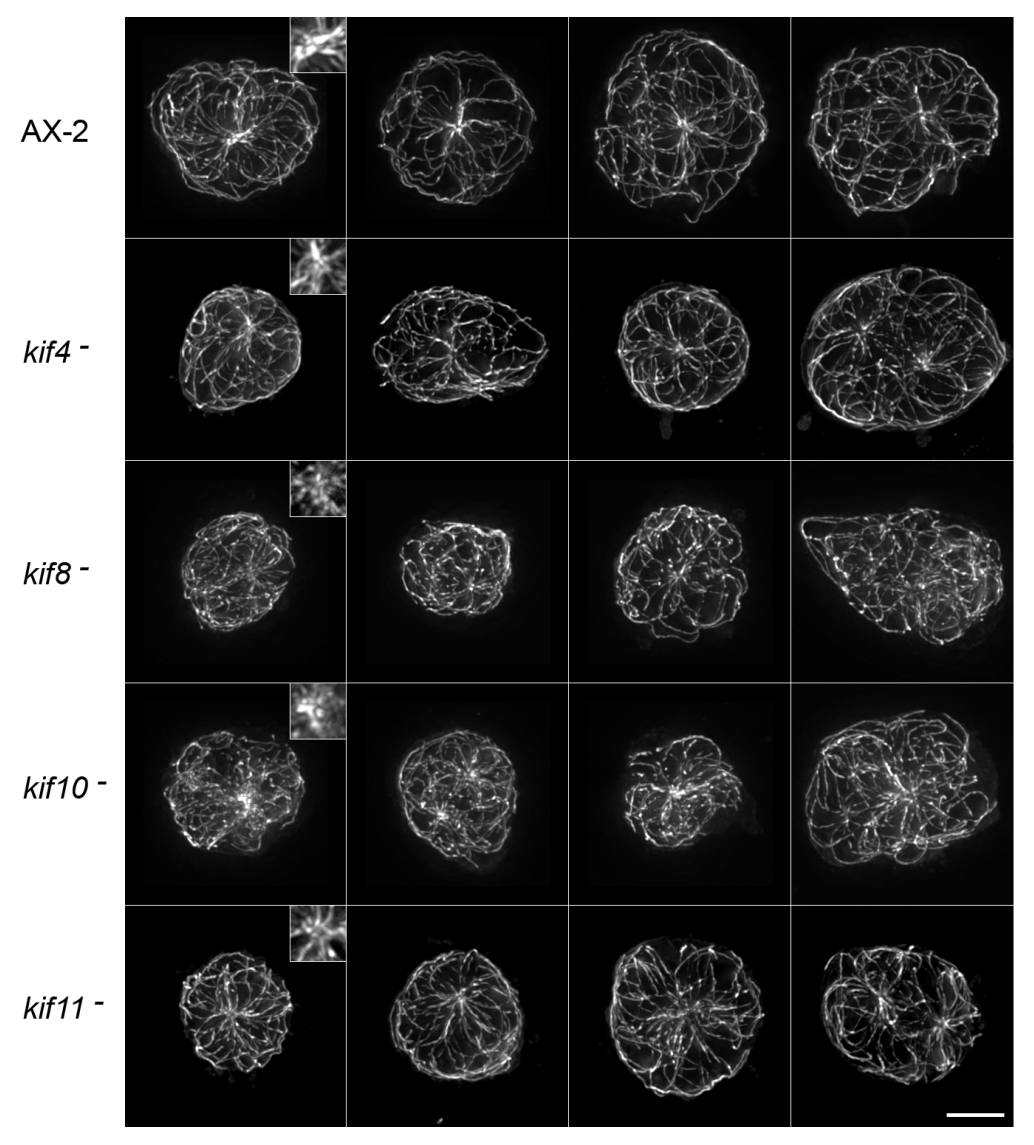
**Microtubule Patterns in Interphase Cells**. Maximum intensity projections of deconvolved image stacks showing interphase microtubule distributions in wild-type AX-2 and kinesin-null cells (fixed cells imaged by indirect immunofluoresence, using a tubulin antibody). Each row displays four examples of the cell strain indicated on the left. Inserts in the first column show 2X enlargements of the MTOC area. For AX-2, *kif4*^- ^and *kif11*^- ^cells, a distinctive ring-like appearance of the centrosome can be seen. This feature is less apparent in the *kif8*^- ^and *kif10*^- ^cells, moreover, a convergence of microtubules into the centrosome in the *kif10*^- ^cell is less obvious. Scale bar = 5 μm.

Since these motors interact with and move along microtubules, and since motor activity is responsible for significant microtubule movement in *Dictyostelium*, we stained fixed cells with tubulin antibodies to determine whether deletions led to any aberrant microtubule distributions. Figure 4 shows a gallery of interphase cells from wild-type AX-2 cells and from the four kinesin mutant lines. All cells display the conventional radial distribution of microtubules that emanate from a centrally located organizing center (MTOC), and, in the few examples shown of binucleate cells, the two centrosomes remain spatially distinct. Interestingly, MTOCs are less distinct in the *kif8*^- ^and *kif10*^- ^cell lines than they are in wild-type, *kif4*^-^, or *kif11*^- ^cells. MTOC's are present in the individual image slices of *kif8*^- ^or *kif10*^- ^cells, but when summed as projections, the ring like appearance of the *Dictyostelium *centrosome is either less apparent or the microtubules do not seem as tightly focused into this structure (inserts in Fig. 4). There were no obvious morphological defects in mitotic microtubule arrays in any of the four kinesin null strains (not shown), although this qualitative observation should be examined in greater detail (see below).

### Kif8, Kif10, and Dynein Cooperate to Organize Interphase Microtubules

**Figure 5:**
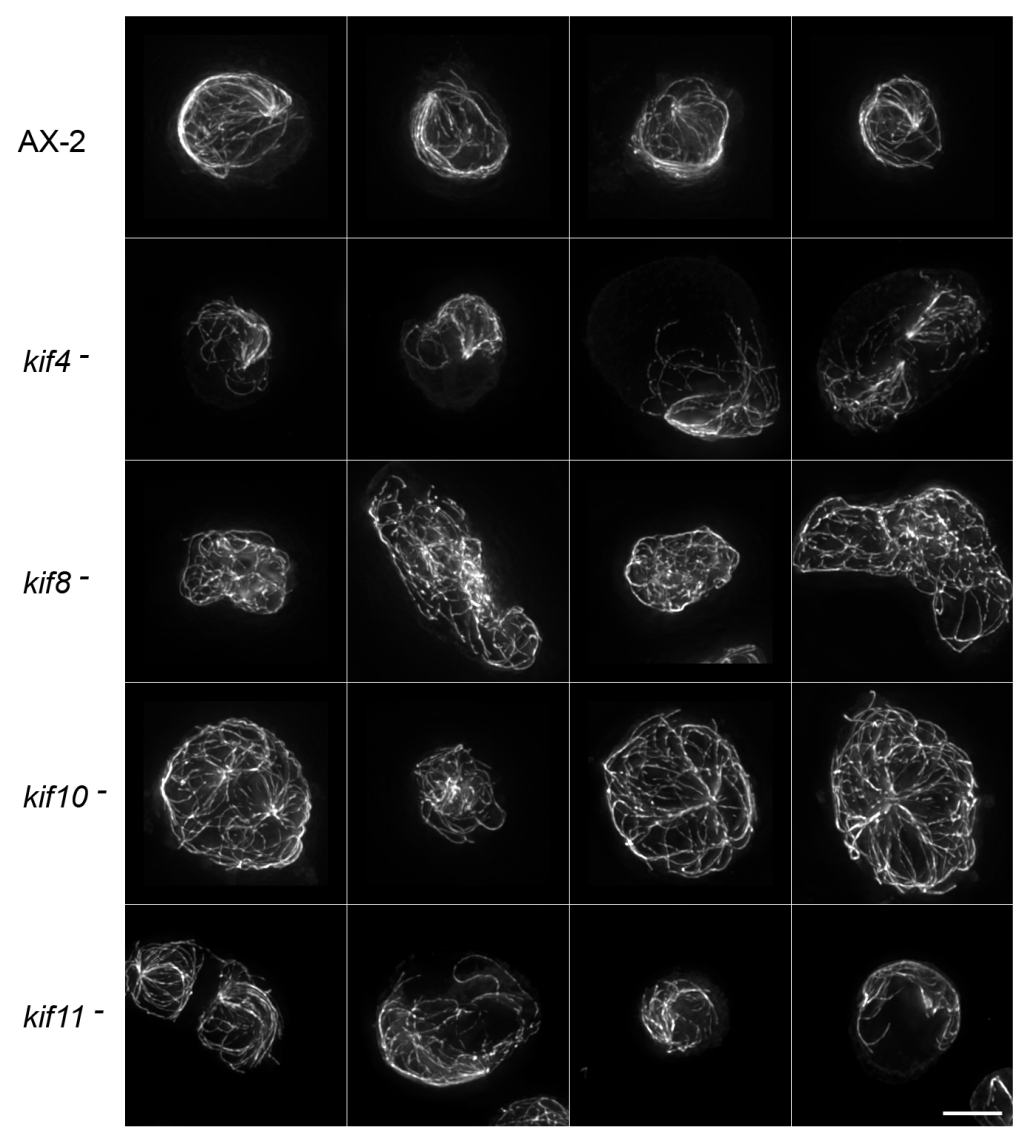
**Microtubule Patterns in Dynein-Inhibited Interphase Cells**. Interphase microtubule distributions, similar to Figure 4 except that each cell line is also transformed with the dynein motor domain expression plasmid (380 K). AX-2, *kif4*^- ^and *kif11*^-^cells display the distinctive 380 K comet-tail phenotype. However, note the relatively normal, radial distribution of microtubules in the *kif8*^- ^and *kif10*^- ^backgrounds. Scale bar = 5 μm.

**Figure 6:**
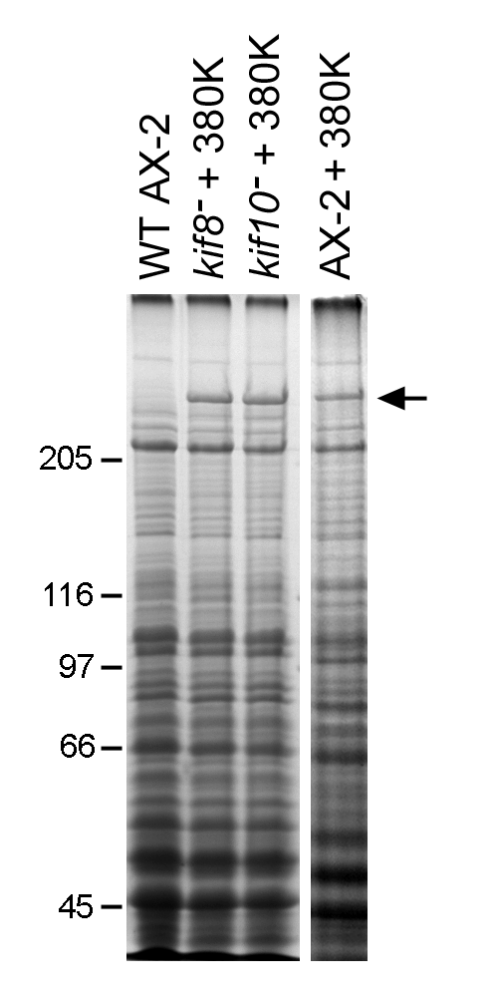
**Expression of the Dynein Motor in *kif8*^- ^and *kif10*^- ^Cells**. Coomassie-stained gel lanes showing high speed supernatants from wild-type control cells, and from *kif8*^-^, *kif10*^-^, and AX-2 cells that have been transformed with the 380 K expression plasmid. The panel confirms the expression of the 380 kDa dynein motor domain polypeptide in *kif8*^- ^and *kif10*^- ^cells (arrow), at a level comparable to the AX-2 cells shown in Figure 5.

We previously characterized a dominant-negative, dynein-mediated defect whereby the entire microtubule network in interphase cells became motile and circulated throughout the cytoplasm (380 K cells) [11,18,19]. The directionality of such motion suggested a role for a kinesin-like motor that pushes against microtubules. To address whether any of the kinesins examined here could have been responsible for generating that aberrant motility, we expressed the 380-kDa dynein motor fragment in each of the four kinesin null backgrounds. The distinctive comet-like microtubule phenotype was found in 82.3% of the control 380 K cells, and in roughly half of the *kif4*^- ^and *kif11*^- ^cell lines (55% and 39.8%, respectively) (Fig. 5, Table 1). However, expression of the 380-kDa polypeptide in either *kif8*^- ^or *kif10*^- ^cells, at levels comparable to affect microtubule organization in control cells (Fig. 6, see also [18,20]), failed to create aberrant microtubule arrays (0%, 4.6%, respectively) (Fig. 5, Table 1). These results suggest that dynein, Kif8, and Kif10 cooperate in producing lateral microtubule motions that organize the interphase microtubule distribution.

**Table 1:** MT Array Morphology in Interphase Cells

Cell-type	Radial MT	Comet-Tail MT	Comets (%)
AX-2 Control	588	19	3.1%
380 K	60	279	82.3%
*kif8*^-^	461	1	0%
*kif8*^-^/380 K	451	0	0%
*kif10*^-^	583	12	2.0%
*kif10*^-^/380 K	475	23	4.6%
*kif11*^-^	212	26	10.9%
*kif11*^-^/380 K	127	84	39.8%
*kif4*^-^	189	9	4%
*kif4*^-^/380 K	68	84	55%

Numbers represent individual fixed cells, either containing a normal, radial interphase microtubule array (Radial MT), or a mutant comet-like microtubule array (Comet-Tail MT) resulting from dynein motor overexpression (380 K) in different control or kinesin null cell stains. % Comet refers to the percentage of total cells that showed the distinctive mutant phenotype.

### Kif4 and Dynein Cooperate in Mitotic Spindle Assembly

**Figure 7:**
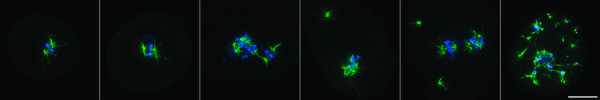
**Spindle Morphologies in *kif4*^-^/380 K cells**. Representative examples of normal and aberrant mitotic spindles in *kif4*^-^/380 K cultures. The two left-most panels show cells with normal appearing metaphase arrangements, with bipolar spindle MTs (in green) that flank condensed chromosomes (blue). These two figures are indistinguishable from mitotic wild type cells. The remaining four panels show examples of aberrant mitotic figures (multipolar spindles, supernumerary or fragmented spindle poles, asynchronous chromatin condensation) that are not normally found in wild-type, *kif4*^-^, or 380 K cells alone. This panel suggests that a combination of dynein and Kif4 (kinesin-7, CENP-E) activity is required for proper spindle assembly. Scale bar = 5 μm.

The *kif4*^- ^deletion is notable, since these cells grow significantly slower than the three other kinesin knockout strains. Interestingly, *kif4*^- ^cells appear normal in shape and size, indicating that the slow growth is not the result of structurally defective cell division (karyo- or cytokinetic failure). When *kif4*^- ^cells were transformed with the 380-kDa expression plasmid, the cell growth rate was reduced even further. While cells grown on a solid surface gradually increased in number, suspension cultures of *kif4*^-^/380 K cells remained at their initial cell density, even when measured for as long as a week. Although defective mitotic figures were not evident in the kinesin-alone knockouts, there was a significant increase in aberrant spindle formation in *kif4*^-^/380 K cells. 66% of the observed mitotic *kif4*^-^/380 K cells (10/15) showed division defects of various types, including multi-polar spindles and supernumerary or fragmented centrosomes (Fig. 7). Although normal-appearing spindles were found among the cell population, it is probable that defective spindle assembly plays a major role in the decreased growth rate of these cells. The results described here indicate that Kif4 and dynein cooperate in some aspect of mitotic spindle assembly; closer evaluation of cell division in these cells is in progress.

## Discussion

We have presented gene deletions for four of the 13 kinesin family members in *Dictyostelium*, and have described the effects of these deletions on cell growth and viability. Individually, none of the four gene products is essential for cell viability nor do the proteins play critical roles in this organism's ability to undergo chemotaxis or to develop upon starvation. The knockout strains do, however, show subtle defects suggesting that many of the key forms of intracellular motility essential for *Dictyostelium *biosynthesis and reproduction are supported by more than one motor protein.

In wild-type *Dictyostelium *cells, both plus end-directed microtubule pushing, and minus end-directed pulling forces are important for maintenance of centrosome position and the radial distribution of interphase microtubules [18,21]. If minus end-directed dynein motility is impaired, a kinesin-like activity appears to dominate and push both the centrosome and microtubule array throughout the cytoplasm [19]. Here we have identified two kinesins, *kif8 *(kinesin-4 family) and *kif10 *(kinesin-8 family), that appear to collaborate with dynein in this organization process. In other eukaryotic cells, kinesin-4 motors participate in a number of diverse activities [22]. One subset of kinesin-4 family members (KIF4) function during mitotic events, with chromatin- and spindle-associated motors that organize bipolar microtubule assemblies and facilitate chromosome alignment [23]. Other subsets of kinesin-4 motors (e.g., KIF21) appear to power interphase organelle transport in cultured cells such as fibroblasts and post-mitotic neurons [24,25]. The single *Dictyostelium *kinesin-4 (*kif8*) is a divergent member of this family, the motor domain is most closely homologous with KIF4 subfamily, yet it contains carboxy-terminal WD-40 repeat motifs in the heavy chain tail that are characteristic of the KIF21 subfamily [3,22]. The kinesin-8 family of motors (*kif10 *in *Dictyostelium*) is thought to mediate chromosome movements through a combination of translocation and microtubule depolymerization activities (recently reviewed in [26], see also [27,28]. The *S. cerevisiae *isoform (Kip3) has previously been shown to cooperate with dynein in positioning mitotic spindles through cortically mediated force production and through control of microtubule length [27,29,30]. Deletions of kinesin-8 isoforms in *Schizosaccharomyces pombe *also suggest a combined force and length control mechanism that positions nuclei and spindles through microtubule-cortex interactions [31,32]. In the absence of either kinesin-4 or kinesin-8 in *Dictyostelium*, we are unable to induce the distinctive centrosome movements via dynein motor overexpression. It is conceivable that Kif8 and Kif10 counterbalance dynein-mediated forces through force-production or anchoring activities at the cell cortex (e.g. kinesin-8) and via lateral microtubule-microtubule interactions (e.g. kinesin-4) that supply sufficient rigidity to allow plus end-directed motors to effectively push (and not simply bend) microtubules. In wild-type *Dictyostelium*, the balance between opposing dynein and kinesin motor activities serves to reinforce the centrosome position and help maintain the radial character of the interphase microtubule array as these cells crawl around and change shape.

Disruption of the kinesin-7 motor (CENP-E) in the mouse is embryonic lethal [33]; this motor is thought to be essential for the proper connection between kinetochores of condensed chromosomes and the mitotic spindle [34]. In contrast, neither member of the kinesin-7 family in *Dictyostelium *(Kif4, Kif11) is essential for mitosis, although removal of Kif4, the isoform that is most homologous to the vertebrate kinetochore CENP-E greatly affects cell growth rate. Preliminary characterization of Kif4 suggests that this motor functions together with dynein in organizing spindle assembly during cell division. While the motor domain of Kif11 is homologous with the kinesin-7 family [3], this polypeptide is significantly shorter and expressed at a much higher level than other CENP-E-like proteins. Outside of a minor enhancement of stationary phase cell density, removal of this motor has no obvious effect on cell viability or function. Closer inspection of each kinesin, and of cells lacking their expression will be required before we can fully understand their individual function(s)

Our study here extends previous work from several laboratories that, taken together, have individually deleted 10 of the total 13 kinesins in *Dictyostelium *[7,9,10,12-14]. All of these deletions have proven to generate cell lines that can survive over multiple generations of growth, indicating that none of these 10 kinesin motors is immediately required for cell viability. Although the Kif12 disruption (kinesin-6, MKLP) produced significant defects in cytokinesis, mutant cells were still able to undergo some form of division that allows strain propagation [7]. The only, potentially essential, kinesin gene reported so far in *Dictyostelium *encodes one of the organelle transporter motors, *kif3 *(kinesin-1 family)[35]. Kif3 can be isolated biochemically and shown capable of powering microtubule gliding, but efforts by Röhlk et al, [35] and in our own lab (Nag, Tikhonenko, and Koonce, unpublished) have not yet yielded viable cells lacking this motor. The resiliency of *Dictyostelium *to motor disruptions is similar to systematic analyses of kinesin isoforms in *S. cerevisiae*, where all six kinesin-related motors (and one dynein isoform) can be individually deleted without loss of viability [5]. The yeast work provided a major guiding principle, for it was the first to suggest that high degree of functional redundancy is present among kinesin family members, and that deletion of motor combinations is required to inhibit cell division. Although, to our knowledge, complete survey disruptions have not yet been reported in other simple eukaryotes, there are clear indications of motor redundancy in some cell models such as *S. pombe *[36], *Aspergillus nidulans*, [37]and *Ustilago maydis *[38]. The kinesins in *Dictyostelium *likewise possess overlapping functions.

The evolutionary transition from very simple single-celled eukaryotes into metazoans generally correlates with an increased number of gene family members. *S. cerevisiae *contains 6 kinesin genes [5]; *S. pombe *has 8 [38]; *U. maydis *has 10 [38]; and *A. nidulans *has 11 [37]). In contrast, even the primitive metazoan *Caenorhabditis elegans *has 21 kinesin genes [39]; *Drosophila melanogaster *has 25 [40], and the human genome codes for 45 kinesins [6] (obviously there are exceptions to this general trend, e.g *Giardia lamblia *contains as many as 23 kinesin genes [41]). One might expect that larger gene families enhance the opportunity for redundant mechanisms, but at least for kinesins, the opposite seems to hold true (summarized in Table 2). Despite the greater number of motor sequences, human cell lines appear far more sensitive to disruption of individual kinesins than do yeast or *Dictyostelium*. Cell division and organelle transport is likely more complex in metazoans, entailing open nuclear spindles, strict spindle position and orientation requirements, cell-type specificities, and many transport features that are not required in unicellular organisms. Thus one could argue that in metazoans, each kinesin motor is custom built for one selective function while in simpler eukaryotes, the motors retain a more generic ability to power microtubule-based transport in a variety of contexts. Understanding the functional details of the same kinesin ortholog in different organisms will therefore define sequence motifs that lead to essential and non-essential functions of basically the same engine, and thus will lead to a more complete understanding of how the motor protein operates.

**Table 2:** Mitotic kinesin disruptions in simple eukaryotes vs metazoans.

**Kinesin Family**	***S. cerevisiae***	***Dictyostelium***	***C. elegans***	***Drosophila***	**Human**
Mitotic Function					
Kinesin-4 (Chromokin)		Kif8	*Klp-19*	*Klp3A*	*Kif4A, Kif4B*
Kinesin-5 (BimC/Eg5)	Cin8, Kip1	Kif13	BMK1	*Klp61F*	*Eg5*
Kinesin-6 (MKLP)		**Kif12**	**Zen-4**	**Pavarotti**	**MKLP1, MKLP2**
Kinesin-7 (CENP-E)	Kip2	Kif4, Kif11		*CENP-E Meta*	*CENP-E*
Kinesin- 8 (Kip3)	Kip3	Kif10		*Klp67A*	*Kif18*
Kinesin-13 (MCAK)		?	*Klp-7*	*KLP10A*	*Kif2A, 2B, MCAK*
Kinesin-14 (NCD/Kar3)	Kar3	Kif2	*Klp-3,15,16,17*	*Ncd*	*KifC1*
Other Function					
Kinesin-3 (Unc104)		Kif1			*Kif14*
Kinesin-10 (Nod)				*Nod*	*Kid*
Kinesin12 (Xklp2)			*Klp-18*		

Normal text (viable), italicized text (not viable) differentiates the individual kinesin-isoform disruptions (knockout, knockdown, or mutation) and their effects on mitosis. The bold text for the kinesin-6 family members indicates cytokinesis defects. In this case, cells can proceed through one or more divisions, but longer term, the mutated protein is essential for organism viability. References for *S.c *[5], *D.d*[7-10, 12-14], *C.e *[39, 47-51], *D.m *[40], and *H.s *[15, 52].

## Conclusion

Analysis of the kinesin gene family in *Dictyostelium *suggests that a significant level of functional redundancy or overlap exists among the organism's motor activities. This result is similar to findings from functional analyses performed in basal organisms such as yeast and fungi, but it contrasts sharply with the roles of individual motors in metazoans. At first glance, most of the kinesins in *Dictyostelium *can be deleted individually without penalty to growth or viability. Yet, upon closer scrutiny or in cases where we impose under additional stresses, we can discern clear phenotypic changes in the cell that provide insight into motor function that may not be obvious in other organisms. Given its greater complement of motor isoforms, and its greater utility of microtubule function relative to other basal eukaryotes, *Dictyostelium *offers an interesting model in which to investigate functional interactions and the regulation of multiple motor proteins.

## Methods

### Molecular Genetics

Kinesin gene sequences were obtained from the dictybase website (see Availability and requirements section). The following primer combinations were used to amplify kinesin gene fragments from AX2 cell genomic DNA; also listed are the downstream kinesin gene-specific primers used for screening recombinants:

*kif4 *(DDB0191404)

Forward: 5'CGC**AAGCTT**AGCCACCAAGACCATTACTTGGACCA 3' (-501 to -476)

Reverse: 5'CGC**GAGCTC**TTAAACTACCACCAATTATTGCGTCATT 3' (+1318 to +1345)

Screen: 5'CATCATCATCCTCTTCACCACTACTATT 3' (+1501 to +1528)

*kif8 *(DDB0191403)

Forward: 5'CGC**GGATCC**GGGTTGCATTAAGAGTTAGACCC 3' (+44 to +66)

Reverse: 5'CCC**AAGCTT**GAATCGGCAGGACTAACACATGC 3' (+ 1302 to +1324)

Screen: 5'GATTGGTTAATACACACCTAATTG 3' (+1381 to +1404)

*kif10 *(DDB0215386)

Forward 5'CGC**GGATCC**TGATCAATATGCAACTCAAGAAGAAG 3' (+249 to +274)

Reverse 5'CCC**AAGCTT**GATCATTGTCATCATCATCATC 3' (+1408 to +1429)

Screen: 5'GTATCATTGATTCATCATTATCCCT 3' (+1501 to +1525)

*kif11 *(DDB0201556)

Forward: 5'CGC**GGATCC**GAATGAACGAGAATATATCGGTTAGC 3' (-2 to +24)

Reverse: 5'CCC**AAGCTT**CCATTACCACTACCACTACCACCT 3' (+1497 to +1520)

Screen: 5'TGACTTGGTGAAACAAATGTTGATC 3' (+1532 to +1556)

+1 of the numbering scheme refers to the position A of the ATG start codon. Restriction enzyme sites were engineered into the ends of each primer (*BamH*1, *Hind*III or *Sac*1, shown in bold type) to facilitate cloning of the amplified DNA into a pUC19 host plasmid, and (in most cases) to excise the DNA construct for transformation. Each construct was sequenced to confirm the identity of the kinesin fragment. Native restrictions sites (Fig. 2) were used to excise and replace an internal fragment of the kinesin sequences (47–669 bp) with a 1.6-kb blasticidin resistance cassette (*Bsr*^*r*^) (*Sma*I digest) from pLRBLP [42], obtained from the *Dictyostelium *Stock Center (see Availability and requirements section for URL). Final constructs were again sequenced to determine the orientation of the *Bsr*^*r *^cassette (diagramed in Fig. 2). The *kif8 *construct was designed to terminate message coding at S202; *kif10 *at N223; *kif11 *at S151; and *kif4 *at W45. In all cases, these disruptions occur upstream of the microtubule-binding domain of the motor.

Standard molecular biology procedures were followed for DNA isolation, manipulation, and blotting. RNA was isolated using the RNeasy kit from Qiagen, following the manufacturer's instructions. *kif8, kif10*, and *kif11 *blots were probed with ^32^P-labeled DNA. the *kif4 *Southern blot was performed using chemiluminescence procedures (ECL, Amersham Biosciences). All blots (Southern and Northern) were probed with the initial amplified genomic target corresponding to the relevant kinesin clone, as indicated above and in Figure 2A.

### Cell Transformation

A calcium phosphate procedure was used to transform *Dictyostelium *AX-2 cells, with 15 μg of linearized DNA per near confluent 10-cm dish (10^7 ^cells) [43]. Transformants were selected with 5 μg/ml blasticidin. Individual colonies were picked with a pipette into 24 well plates, and were screened by PCR for homologous recombination. Amplification of a 1.6-kb target with a primer internal to the *Bsr*^*r *^marker (5' GAATGGCAAGTTAGTCAAAACTACG 3') and a primer downstream of the recombination site (indicated above for each kinesin sequence) was used to initially identify positive recombinants. Cells from positive colonies were further purified by serial dilution, and were again confirmed by PCR with downstream and upstream primer combinations. For dynein disruptions, we introduced a motor domain expression plasmid (aa 1384–4725), into kinesin null cells by either a CaPO_4 _or an electroporation method [44]. *kif*^-^/380 K expressing cells were selected with 10 μg/ml G-418 (geneticin, Sigma Chemical Co).

### Light Microscopy

Cells were flattened on glass coverslips using an agarose sheet, fixed with formaldehyde, labeled with a tubulin antibody [45], and in some cases Hoechst 33342, as described in [11]. Z-series of images were obtained on a DeltaVision light microscopy workstation and were deconvolved using softWoRx 2.5 (Applied Precision, Issaquah, WA). Maximum intensity projections were compiled using ImageJ (NIH); figures were assembled in Adobe Photoshop. For cell growth measurements, triplicate 100-ml cultures were seeded with 9 × 10^4 ^cells/ml, shaken at 200 rpm at RT, and counted with a hemocytometer every 24 hr. Growth curves were calculated and displayed with Microsoft Excel; error bars indicate standard deviation.

## Availability and requirements

The Dictybase website: 

Dictyostelium Stock Center: 

## Authors' contributions

DKN designed the knockout constructs and participated in the molecular genetic studies. IT participated in the gene knockout and screening work, performed cell culture analyses and 380 K dynein expression analyses. IS participated in the molecular genetic analysis. MPK performed the light microscopy, participated in the molecular genetic studies, assembled the figures, and wrote the manuscript. All authors read and approved the final manuscript.
